# Supplementary material for: Identification of photoperiod-induced specific miRNAs in the adrenal glands of Sunite sheep (Ovis aries)
Source: Front Vet Sci. 2022 Jul 22;9:888207. doi: 10.3389/fvets.2022.888207 (PMC9354845; doi:10.3389/fvets.2022.888207)
Supplement: Supplementary file 1 [file Data_Sheet_1.ZIP › Supplementary file/miRDeep2 file.zip!/miRDeep2 file/Know_Structure/SP42A2/oar-mir-410.pdf]

miRBase precursor : oar-mir-410
Total read count : 1290
oar-miR-410-5p read count : 8
oar-miR-410-3p read count : 1276
remaining reads : 6

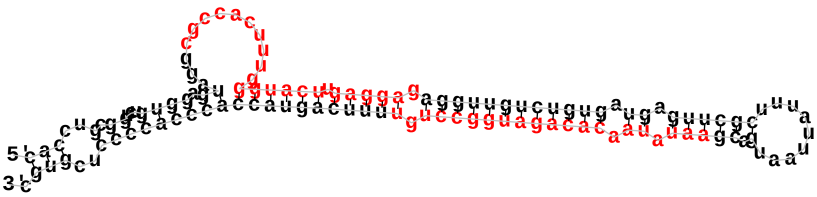

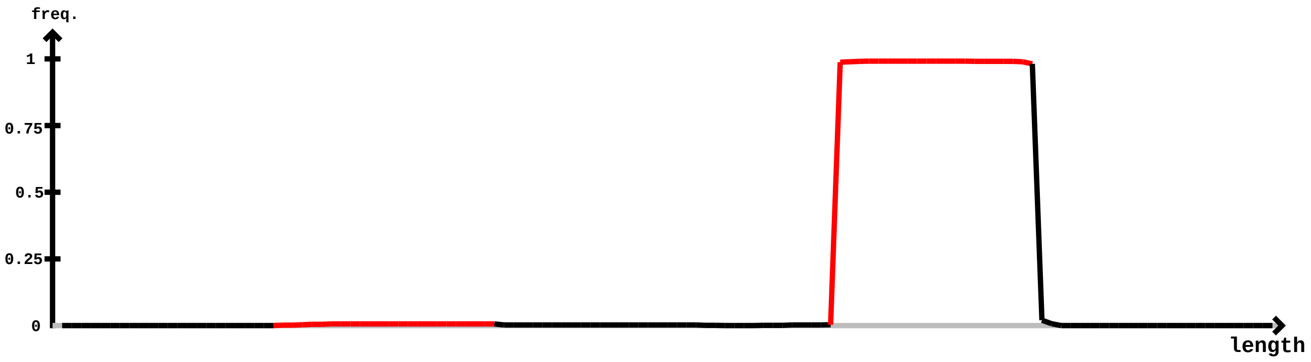

oar-miR-410-5p

oar-miR-410-3p

```
5'- caccugcggucuguggaguaggcgccacuuuggguacuugaggagagguugucugugaugaguucgcuuuauuaaugacgaauauaacacagauggccuguuuucaguaccacccaccccucgugc   -3'    exp
    (((..(.(((..((((........))))....((((((.(((((.((((((((((((.((.((((((.........).))))).)).)))))))))))).))))))))))....))).)..))).      reads      mm
    ......................cgccacuuuggguacuugaggag.................................................................................      2          0
    ........................ccacuuuggguacuugaggag.................................................................................      2          0
    .........................cacuuuggguacuugaggag.................................................................................      2          0
    ...........................cuuuggguacuugaggag.................................................................................      2          0
    .............................................agguugucugugaugaguucg............................................................      2          0
    .............................................agguugucugugaugaguucgcu..........................................................      1          0
    ........................................................................uaaugacgaauauaacacagau................................      1          0
    ...........................................................................ugacgaauauaacacagauggccugu.........................      1          0
    ...........................................................................ugacgaauauaacacagauggccuguuu.......................      1          0
    ...............................................................................gaauauaacacagauggccugu.........................      1          0
    ................................................................................aauauaacacagauggccu...........................      2          0
    ................................................................................aauauaacacagauggccug..........................      10         0
    ................................................................................aauauaacacagauggccugu.........................      1236       0
    ................................................................................aauauaacacagauggccuguu........................      17         0
    ................................................................................aauauaacacagauggccuguuu.......................      5          0
    .................................................................................auauaacacagauggccugu.........................      1          0
    .................................................................................auauaacacagauggccuguuu.......................      1          0
    ..................................................................................uauaacacagauggccugu.........................      1          0
    ..................................................................................uauaacacagauggccuguuu.......................      1          0
    ...................................................................................auaacacagauggccuguuu.......................      1          0
```
